# EHMTI-0016. Examples of problematic use of references in the headache literature. A problem for readers, authors, referees, and editors ?

**DOI:** 10.1186/1129-2377-15-S1-D65

**Published:** 2014-09-18

**Authors:** P Tfelt-Hansen

**Affiliations:** 1 Department of Neurology, Danish Headache Center, Glostrup, Denmark

## Background

The problem with references is incorrect use to support statements in the text. This will be illustrated by presenting two of many observed examples.

## Methods

Uncontrolled personal observations of dubious references in the headache literature. Examples:

Statement: Nagata et al. (4) reported that spontaneous migraine was not associated with any dilation of the middle meningeal artery as measured by MRA.

Reference: 4. Nagata E, et al. The middle meningeal artery during a migraine attack: 3T magnetic resonance angiography study. Intern Med 2009; 48: 2133–2135.

Comment: Note that only one migraine attack was studied.

Statement (from a kinetic study on orally inhaled DHE): Other routes of administration such as nasal delivery (40% bioavailability) have erratic and somewhat unpredictable pharmacokinetic (PK) properties that pose therapeutic challenges (eg, unpredictable clinical response or adverse events)(4)

Reference: 4. Saper JR, Silberstein S. Pharmacology of dihydroergotamine and evidence for efficacy and safety in migraine. Headache 2006;46 (Suppl 4): S171-81(in this reference no mention at all of inconsistent kinetics of nasal DHE).

Comment: The statement is from an industry-sponsored paper on kinetics of the new orally inhaled DHE and discredits to some extent nasal DHE. However, an extensive study of nasal DHE from 1996 demonstrated “the reliability and reproducibility” of nasal DHE’s pharmacokinetics (Humbert H, et al. Human pharmacokinetics of dihydroergotamine administered by nasal spray. Clin Pharmacol Ther 1996; 60: 265–275).

## Conclusion

Problematic references should not be used neither by scientists nor by the industry. Some initiatives to diminish the problem will be suggested.

No conflict of interest.

